# Participation of African social scientists in malaria control: identifying enabling and constraining factors

**DOI:** 10.1186/1475-2875-3-47

**Published:** 2004-12-06

**Authors:** Paulyne M Ngalame, Holly Ann Williams, Caroline Jones, Isaac Nyamongo, Samba Diop, Felisbela Gaspar

**Affiliations:** 1 Master of Public Health Program, Morehouse School of Medicine, 720 Westview Drive, SW., NCPC Suite 344, Atlanta, Georgia, 30310, USA; 2 Malaria Branch, Centers for Disease Control and Prevention, Mail Stop F-22, 4770 Buford Hwy, NE Atlanta, GA, 30341, USA; 3 Department for International Development (DFID) Malaria Programme, London School of Hygiene and Tropical Medicine (LSHTM), Keppel Street, London WC1E 7HT, UK; 4 Institute of African Studies, University of Nairobi, P.O. Box 30197, Nairobi, Kenya; 5 Department of Public Health, Epidemiology and Medical Anthropology, University of Bamako, BP 1805, Bamako, Mali; 6 National Institute of Health, P.O. Box 264, Maputo, Mozambique

## Abstract

**Objective:**

To examine the enabling and constraining factors that influence African social scientists involvement in malaria control.

**Methods:**

Convenience and snowball sampling was used to identify participants. Data collection was conducted in two phases: a mailed survey was followed by in-depth phone interviews with selected individuals chosen from the survey.

**Findings:**

Most participants did not necessarily seek malaria as a career path. Having a mentor who provided research and training opportunities, and developing strong technical skills in malaria control and grant or proposal writing facilitated career opportunities in malaria. A paucity of jobs and funding and inadequate technical skills in malaria limited the type and number of opportunities available to social scientists in malaria control.

**Conclusion:**

Understanding the factors that influence job satisfaction, recruitment and retention in malaria control is necessary for better integration of social scientists into malaria control. However, given the wide array of skills that social scientists have and the variety of deadly diseases competing for attention in Sub Saharan Africa, it might be more cost effective to employ social scientists to work broadly on issues common to communicable diseases in general rather than solely on malaria.

## Introduction

Malaria control remains ineffective in many endemic areas in spite of efficacious interventions, such as combined antimalarial therapies and insecticide-treated materials. Biological, environmental, political, socio-cultural, economic and behavioural factors influence the transmission of malaria, thus requiring a multidisciplinary and integrated approach to effectively control the spread of malaria [1,2].

In recent years international initiatives, such as Roll Back Malaria, have highlighted the contributions that social scientists bring to a multidisciplinary approach for malaria control. Various studies in Africa have illustrated the benefits of collaborative research between social and biomedical scientists [3-17]. However, social science knowledge and practices are still not fully integrated into malaria research and control programmes, especially when compared to other public health areas such as HIV/AIDS prevention. While it is difficult to do a comparison on the actual number of social scientists involved in HIV/AIDS versus malaria control, a review of the literature illustrates the numerous contributions that social and behavioural research has had in HIV/AIDS control and prevention [18-27]. The early recognition and inclusion of socio-behavioural research was part of the HIV/AIDS prevention strategy at the onset of the pandemic [27]. Significant funding allowed social scientists to work directly with epidemiologists in HIV/AIDS research.

Several factors continue to limit the application of social science research in tropical public health in general, and malaria control in particular. These include: confusion among many non-social scientists and social scientists alike about the variations in social science theory and methods and the different types of contributions that each can make to public health and malaria control activities, a perception among many public health and malaria control professionals that the problems of communicable diseases can be solved with technical and clinical interventions, ignoring the social nature of illness, limited communication among social scientists working in applied health and malaria control, insufficient access to current social science and malaria literature, and the lack of trained social scientists with applied experience in the endemic countries [28,29].

Given the poor academic and professional structures found in many African countries, an increasing number of highly skilled citizens emigrate abroad for further studies but fail to return to their respective country of origin, a phenomenon commonly referred to as 'brain drain' [30-36]. As a result, many African countries now have a vast pool of highly skilled professionals who are permanently living abroad, making little to no contributions to their country of origin [37,38]. While the "brain drain" significantly contributes to the lack of social science capacity in malaria control in Africa, it is unclear the extent to which the current pool of trained social scientists that remain in Africa is being integrated into malaria control. Experience has shown that commissioning a well-trained and field-experienced applied social scientist (i.e. one with both an understanding of the theoretical and applied perspectives of the discipline) can be extremely beneficial for informing malaria programme-related decisions, as well as helping in the development of effective intervention programmes [39,40]. This study was designed to better understand the level of involvement of African social scientists in malaria control, in order to identify potential approaches to facilitating collaborative work among social scientists and malaria control programme personnel and stakeholders.

## Methods

A convenience sample was derived from existing social science networks such as, the Partnership for Social Science and Malaria Control (PSSMC) Network, the Social Science and Medicine Africa Network (SOMA-Net), Pan African Anthropological Association (PAAA), as well as the CHANGE Project database of African social scientists, and the United Nations Development Program, World Bank and World Health Organization's Special Programme for Research and Training (WHO/TDR) database of social scientists trained in the last decade (1992–2000). Snowball sampling was used to elicit names of additional social scientists known personally to the participants and investigators.

Social science was broadly defined to include disciplines such as anthropology, sociology, health economics, demography and population studies, development studies and public health. Enrolment criteria included specific social science training within these disciplines, and participants had to be of African origin. Enabling factors were defined as those factors that made it easy to work in malaria control, including factors that first attracted social scientists and those that facilitated entrance into the field. Constraining factors were defined as those factors that impeded work in malaria control and factors that made malaria control unattractive to social scientists.

Data collection was organized in two phases. In Phase I, consent forms and questionnaires were sent by email and/or fax to potential participants. Participants could respond directly and/or refer other eligible colleagues. Those who were ineligible or uninterested could decline. For those emails that could not be delivered, three attempts were made to resend the survey and consent form. The questionnaire focused on demographic characteristics, work experiences, factors influencing career choices and sources of career development information. The survey instrument was piloted among five participants from different countries. Based on the feedback from the pilot survey, minor adjustments were made to improve use via email. Email communication was preferred but, in the absence of email, other methods of communication were used. If the survey was not returned within three weeks, attempts were made to follow-up.

The survey responses in Phase I provided an overview of the involvement of the participants in malaria related activities but gave only a limited understanding of the specific factors affecting career trajectories. In Phase II, we conducted in-depth phone interviews using a purposive sample derived from Phase I that was selected according to country of origin, sex, academic field (social science discipline) and interest in malaria and applied or operational work experience (both in malaria and non-malaria). A deliberate effort was made to ensure that the sample represented those with or without an interest in malaria, those who actively sought employment in malaria and those who did not.

The more descriptive data from Phase II clarified Phase I data. For example, with regards to participants' interest in malaria, some participants in Phase I indicated an interest in malaria while in school, but did not include malaria in their thesis research and did not pursue employment in malaria. The in-depth, open-ended phone interviews in Phase II provided greater detail as to other factors that might have influenced participants' career decisions in malaria control. Open-ended questions focused on key events and times in the career trajectory during which critical decisions were made, current and past work experiences, reasons for selecting specific jobs, technical experiences with proposal writing, challenges and rewards of working in malaria, and suggestions for improving the integration of social science in malaria control.

Descriptive statistics were generated using EPI-Info. Qualitative data were transcribed and content analysis was used to develop contextual themes. The study was reviewed and approved by the Institutional Review Board of the Centers for Disease Control and Prevention. Verbatim quotes from the participants are used throughout the text to illustrate contextual points of discussion.

## Results

**Figure 1:**
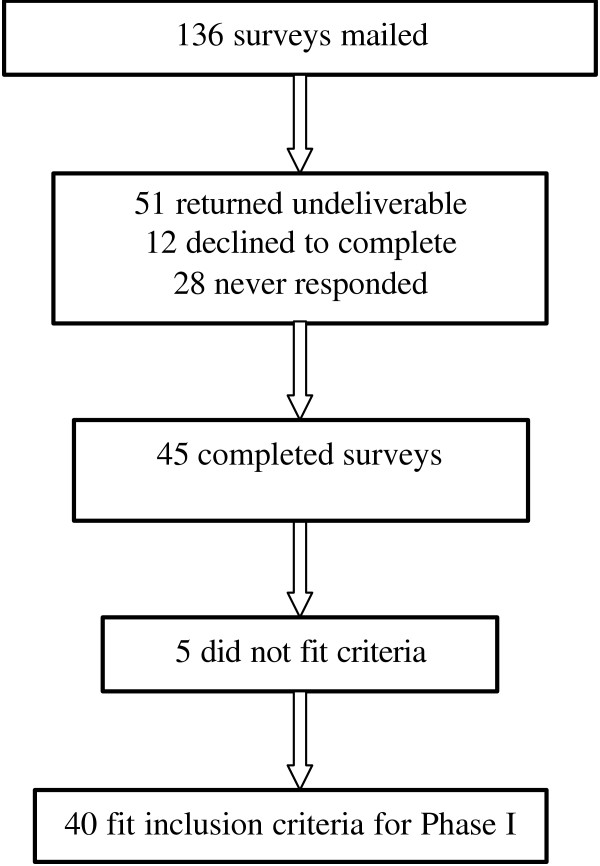
Response to surveys. Flow chart illustrating response to surveys

This study was conducted between May 2002–May 2003. Of the 136 surveys sent out, 40 completed surveys were found to fit our enrolment criteria (Fig. 1). Eighteen participants were interviewed in Phase II.

All 40 participants were nationals of African countries and resided in the countries of Cameroon, Ethiopia, Mali, Ghana, Kenya, Nigeria, Tanzania, South Africa and Uganda. Most participants (75%) were male between the ages of 30–39 years old (Table 1). Fifty percent (n = 20) had a masters degree, 42% (n = 17) were either doctoral degree students (n = 3) or had a doctoral degree (n = 14). Almost all participants had an undergraduate degree in the social sciences and chose various social sciences disciplines for specialization during their postgraduate training.

**Table 1:** Age and Sex Distribution (n = 40)

Age group	Male	Female	Percentage
20–29	1	0	2.5%
30–39	17	5	55.0%
40–49	9	4	32.5%
50–59	2	1	7.5%
60+	1	0	2.5%
Total	30	10	100.0%

Of the 18 participants selected for a phone interview, 55% (n = 10) were between 30–39 years and 22% (n = 4) were female. Of those with postgraduate degrees, 50% (n = 9) had a masters degree, 44% (n = 8) either had a doctoral degree or where doctoral degree students while 5% (n = 1) had a bachelor's degree. When difficulties arose in ascertaining whether the professional discipline linked to the social sciences, participants were contacted for clarification.

Eighty-five percent of the participants (34/40) received funding for postgraduate training from their national government, international organizations (such as the WHO or DFID), or other foreign governments. Sources of career development information included the Internet, friends and colleagues, local universities, conferences, journals, professional networks and local newspapers.

### Enabling factors

#### Interest in malaria

When asked if participants were interested in malaria during their academic training, 82% (33/40) of the participants said yes. Only 36% (12/33) of those interested included malaria as a research topic in their thesis or dissertation. Three participants (7.5%) who were not interested in malaria included malaria in their thesis. Post graduation, 35% (14/40) of the participants actively sought employment in malaria control. Three of the 14 (21%) who sought employment in malaria were turned down for jobs due to insufficient technical knowledge of malaria control, while six participants (15%) returned to previous health care positions, some of which were positions in malaria control. Although some participants were not currently working on malaria-related projects, nearly all participants, at some point in their career, had worked as a consultant in malaria control in addition to their full time positions. Participants who specialized in malaria for postgraduate degrees were often funded by a national or international organization, on condition that they would return to their jobs in malaria following the completion of studies.

Eleven of the 40 participants said that the primary factor that prevented those with an interest in malaria from seeking further research and employment opportunities in malaria control was the perception that social scientists could not be employed in malaria control. As one participant remarked *"Malaria is an endemic disease in my community, so I can't say I was never interested. I wanted to do a thesis on malaria, but I realized that social scientists could not get jobs in malaria so I had to look elsewhere." *When followed up during the phone interviews in Phase II, it was clear that some participants turned down jobs in malaria due to competing employment in areas such as HIV/AIDS, while others declined malaria control contracts due to the competing demands of doctoral-level training.

Another issue that was revealed in Phase I was the influence of the epidemiology of malaria on participants' interest in malaria. Eleven of the 18 participants during the phone interview explained that they developed an interest in malaria after learning about the complex nature of the disease, its high prevalence and the possibility of access to research funds. *"For years I thought the field of malaria was boring and I was not interested in it. I only became interested in malaria when I realized that it was a complex disease with a major public health impact and it appeared that funding for research might be available."*

#### Factors influencing employment in malaria

All 40 participants were asked to identify factors that attracted social scientists into general employment opportunities, as well as malaria-specific opportunities (Table 2). Responses in Phase II provided further clarification on the specific factors affecting career trajectories. Of the 18 participants included for an in-depth interview in Phase II, 11 indicated that a senior lecturer or mentor during the practical training experience had encouraged participants to work with them on their research project. Mentors helped to develop participants' research and proposal writing skills and identify funding and publication opportunities, and in some cases international contracts. These contracts often resulted in participants gaining local and international visibility within the larger malaria community, which led to subsequent employment opportunities.

**Table 2:** Factors Important for Employment (n = 40)

**Malaria Employment**	**Frequency**	**General Employment**	**Frequency**
Sufficient funds to complete job	11	Potential for career advancement ^**a**^	26
Supportive environment that facilitates the translation of research findings into programmatic use	9	Competitive salary ^**a**^	13
Ability and opportunity to contribute alternative solutions to malaria control from a social science perspective	6	Proximity to family ^**a**^	12
Type of social science dimension to job	5	Social value of job ^**b**^	5
Sufficient technical skills to complete jobs	5		
Epidemiology of malaria	4		

^**a**^Indicates factors that were identified as important for both general employment and malaria specific employment.

^**b**^The social value of a job was defined as the positive impact that a given job made in the community, as well as the professional and community respect awarded to the position. Participants were more likely to seek out employment from organizations with proven track records of using research findings to improve the health of the community.

Opportunistic events also shaped career development. For example, the successful completion of a project, fellowship or consultancy often produced a cascade effect by opening up other opportunities in malaria control, resulting in individual professional recognition by local and international collaborators as an expert in the field. Attending workshops sponsored by international organizations, such as the World Bank and WHO, enabled participants to get their name in the organizations' database for possible consultancies.

Strong writing skills were identified as essential for job and training opportunities by most of the 18 participants in Phase II. Ten of the 18 participants (55%) in the phone interview noted that while it was initially difficult to identify and develop fundable proposals, receiving constructive feedback from rejected proposals helped improve their writing skills and led to the development of more successful proposals. *"Most of the rejected proposals do not give reasons for rejection, which is unfortunate. I have been lucky to have one rejected proposal that was returned with explanations as to why it was rejected. I was told the issue we raised was very broad and vague and it made me realize that I had to be very clear in my framework. After that rejection, I was able to develop projects that had a clear framework and include a multidisciplinary team to strengthen other key areas and the next proposal we developed was accepted."*

When asked why they chose employment in malaria, one of the participants echoed many others by saying, *"In Africa we take what we get. One rarely ends up in a profession of their choice. People take the job that is at hand and learn to deliver as best as they can." *This was true for both those interested in malaria originally and those not interested. During the in-depth interviews, we also asked participants who did not initially express an interest in malaria but sought and gained malaria related employment, why they choose to stay in malaria? Of the 18 participants eight agreed with the following view: *"I would probably stay in malaria because it is a major problem in my country, and I feel I have gained significant experience in malaria over the years. This has strengthened my confidence and ability to handle current and hopefully future opportunities, and maybe I am too old right now to start learning something new."*

### Constraining factors

#### Malaria employment challenges

Participants were also asked to identify factors that constrained employment in malaria. All 40 participants were more likely to refer to employment challenges specific to malaria research, rather than malaria control, as national malaria control programmes employ very few social scientists. Ten of the 18 participants in Phase II noted that social science involvement in malaria control was a recent development in their countries, as biomedical scientists had previously dominated malaria control. Another phone interview participant remarked that *"specifically focusing on malaria as the only area for job opportunities narrows ones fields for job opportunities. Although malaria is an endemic disease in my country, it is largely dominated by physicians and there are not sufficient job opportunities for social scientists in my country so it is impossible to focus only on malaria. You need something to fall back on besides malaria." *Participants also noted that a paucity of research centres and limited funding for social science research also contributed to the lack of job opportunities. In situations where job opportunities existed for social scientists, they were generally limited to short-term rather than long-term career paths.

Individual perceptions of malaria as a 'normal' everyday event limited participants' understanding of the impact of malaria and, thus, diminished their interest in malaria control. As one of the phone interviewed participants remarked, *"At first, I did not realize the impact of malaria in my community. Everybody was talking about HIV/AIDS. I think it is because people have lived with malaria for so long that they treat it at home. It was only after doing some training at the hospital (as part of the national training requirement), that I realized how many people, especially children and mothers, die of malaria and I got interested in working in malaria control."*

Of the 40 participants in Phase I, 36 identified the lack of professional development opportunities as a constraining factor to employment in malaria.  The lack of a supportive work environment with good communication and mutual respect was mentioned by those working primarily with biomedical scientists.  *"I prefer working on maternal and child health issues, as well as HIV/AIDS, because personnel in these areas are able to recognize the role of social science and request social science input.  This makes me confident that my discipline is important and I can make a contribution." *

Insufficient technical skills in proposal writing and professional isolation from other social scientists hampered the development of collaborative relationships, which made it difficult to develop competitive proposals for funding and publication.  As a participant observed, *"Research is largely uncoordinated.  We have no knowledge of who is doing what, there is no database of social scientists and the projects that they're working on from which we can develop collaborative projects.  We neither have the infrastructure nor the access to current literature, which makes it difficult to develop manuscripts for publication."  *

In order to meet the challenges of employment, eight of the 18 participants in Phase II stressed the importance of understanding all technical aspects of malaria control. One participant echoed the view of several others in their advice to junior social scientists, *"try to learn not only about social science in malaria control, but all other aspects of malaria control as well, such as entomology, vector control and treatment. Having only a social science background without any understanding of the other areas of malaria control might make it difficult to effectively apply the social science aspect within malaria control. Besides you will always be working as part of a multidisciplinary team, therefore, you will need to understand their language in order to be able to effectively communicate the social science dimension to them."*

Participants also emphasized the importance of linking students to practical training opportunities in endemic areas for fieldwork experience and possible long-term opportunities. As one participant noted, *"Encourage donors to develop grants for student researchers working with senior scientists, and identify conferences where students could present research findings."*

### Integrating social science in malaria control

Participants in Phase II were asked to describe strategies for improving the integration of social science in malaria control. Of the 18 participants interviewed, 13 (72%) stressed the need for social science advocacy as an important step in malaria research and control. Advocacy efforts were recommended for social scientists and non-social scientists alike, as well as policy makers in health and education and those who fund research. Five participants said their involvement in malaria projects was as a result of specific requests for social scientists in the call for proposals.

All 18 participants emphasized the need to develop outreach efforts for junior social scientists in order to enhance their understanding of the potential contributions that social scientists could offer, the type of training needed, and the types of jobs they could perform in malaria control. Three of the 18 participants also suggested the revision of graduate and medical school curricula to include malaria and social science courses, but noted that support had to come from the national level for curricula change to take place.

The establishment of a sustainable network of African social scientists working in malaria control was identified as a useful tool for integrating social sciences in malaria control by six of the 18 participants. Possible functions of the network were described as developing a mentoring programme for junior social scientists, a forum to disseminate current local social science research, information relating to employment and training and funding opportunities.

To facilitate the application of social science research findings to national malaria control policies, one of the phone-interviewed participants explained how to include policy makers in the process of social science research. *"Make the research process more participatory. Involve policy makers and consult with them so that eventually they are the ones who demand the research and in this way, social science research can influence policy. We developed working papers with key findings for distribution to various stakeholders. We published our findings on the Internet and conducted department seminars or short courses during which we discussed our research and presented the information in a very manageable manner. At least, in this way, we were able to get our findings into the hands of policy makers and hopefully begin a dialogue with them."*

## Discussion

Results of this study indicate that factors such as having an interest in malaria, a mentor during one's academic and professional career, strong writing skills, and technical experience in malaria made it possible for participants to take advantage of opportunistic events and job opportunities that led to employment in malaria control activities. As well, limited job opportunities, the lack of career development opportunities, and the lack of understanding about social scientists' role in malaria limited the involvement of social scientists in malaria control. This study revealed that many of the enabling and constraining factors for social scientists' involvement in malaria control are not unique to malaria control but are common to African researchers in general. An examination of the intrinsic and extrinsic factors regarding career trajectories of social scientists in this study revealed that the majority of participants did not specifically seek malaria as a career path but, rather, were looking for employment opportunities in general, some of which happened to be in malaria. It is unclear to what extent the career development process in many African countries contributes to the way employment is sought or if this is due to the relatively new role that social science plays in malaria control, which was noted by numerous participants. Career development is a process that ideally begins during one's formal academic training and continues throughout one's professional life. However, if career development is concentrated more towards academic training and less on postgraduate employment opportunities, it is no wonder that post graduation, students are forced to 'take whatever they can get,' as opposed to seeking opportunities that complement their interest and training.

The lack of proper career counselling could also limit a student's understanding of what courses and practical experiences are most beneficial for social science and health careers, without which social scientists cannot be hired as part of a health intervention team [39,41]. More initiatives that provide research-training grants, career development fellowships and proposal development workshops, such as those currently established by the UNDP/World Bank/WHO/TDR, are needed to strengthen the social science capacity in endemic countries. However, creative strategies need to be identified to capture those who do not easily have access to such information and opportunities.

In order to effectively design and implement malaria control interventions, it is necessary to have social scientists that are knowledgeable about the technical issues that underlie malaria control. This knowledge is acquired over time and needs to be supplemented with direct field experiences. However, given the economic constraints facing national malaria control programmes and Ministries of Health, this study shows that it might not be reasonable to expect that social scientists will be assigned solely to malaria control. Given the limited number of trained social scientists with practical experience and the variety of deadly diseases competing for attention in sub-Saharan Africa, there are few social scientists that are both willing and able to dedicate their expertise only to malaria control.

Ministries of Health and Education should work with social scientists to establish employment opportunities in communicable diseases. Given the wide array of skills that social scientists have, it would be cost effective to employ social scientists to work broadly on issues common to communicable diseases, rather than solely on malaria. This "generic" approach would also support the programmatic shift from malaria as a vertical programme to its being integrated within primary health services or the wider arena of communicable diseases. Although this may not be ideal for building strong social science capacity specifically for malaria, this offers a pragmatic approach to utilizing scarce resources. Lessons learned from behavioural interventions in other diseases can also be applied to malaria control, thus strengthening social science contributions to malaria control.

The acknowledgement by many participants of their need for a better understanding of both biomedical and technical aspects of malaria control is reinforced by data from other successful projects that are aimed at establishing collaborative multidisciplinary research teams. For example, the International Clinical Epidemiology Network (INCLEN) social science programme seeks to enhance clinicians' understanding of social science by exposure to relevant issues in social science design and measurement and evaluation issues, while teaching principles of clinical epidemiology and biostatistics to established social scientists. As a result, strong collaborative partnerships among individual clinical epidemiologists, social scientists, and biostatisticians have been developed to produce the interdisciplinary solutions required for priority health problems in society [42].

A key factor identified as important for employment in malaria was the ability to contribute social science solutions to malaria control and the integration of research findings into malaria control programmes and policies. Social scientists are more likely to work directly with members of the community therefore, it is important that strong relationships are developed with local communities and their leaders, as a trust building mechanism and a matter of social prestige for the researcher. This link to the community facilitates the application of research findings on a local level [39,42].

### Limitations

While findings from this study give us greater insight into factors that affect career trajectories of African social scientists, caution must be used when interpreting the results. The use of convenience sampling and snowball sampling techniques to identify eligible participants appeared to have excluded the younger more recently qualified social scientists who might not have been members of the various networks we used to identify eligible participants for this study. It is possible that some of the junior social scientists do not have much work experience in malaria and are not yet known by the more experienced social scientists that helped with referring other participants for the study. In addition, although telephone interviewing enabled us to reach social scientists from several countries, we were not able to reach those social scientists with no access to the Internet and telephone who might have had other unique challenges not captured in this study. Further research should examine younger or more recent social science graduates as well as those with no access to the Internet.

## Conclusion

Malaria control is at a point where there is good evidence-based data to demonstrate that various approaches can make a difference in malaria-related morbidity and mortality. Prompt and timely treatments with an effective drug, intermittent preventive treatment for malaria in pregnancy, and use of insecticide-treated materials are examples of interventions that have been shown to be effective. However, the success of these approaches depends, to a great extent, on human behaviour. Epidemiologists and clinicians, who work in malaria control, traditionally, do not have the necessary training to incorporate behavioural and social science knowledge into the development, implementation, and evaluation of control interventions. It is time to broaden the perspective of malaria control, which can be best achieved through employing a multidisciplinary team that includes social scientists. To support such collaboration, attention needs to be given to understanding the factors that influence job satisfaction, recruitment and retention of the social scientists.

At this point in time, social science capacity is severely limited in most African malarious areas due to insufficient funding, lack of career structure, loss of trained individuals to international postings, and a limited understanding of how social scientists can contribute to malaria control. In order to improve this situation, capacity development is needed as a first step. Joint collaborative efforts should be made to offer technical malaria knowledge to social scientists and social science methodology to epidemiologists and control personnel. Social scientists need forums and networks to exchange information, learn about existing research and educational opportunities and promote collaborative partnerships with national malaria control programmes. However, these mechanisms cannot be realized without adequate levels of funding.

In order to attract skilled social scientists to Ministries of Health, a new line of thinking needs to emerge that situates social scientists more broadly within the ministry. Rather than focusing on how to integrate social scientists solely within malaria, the emphasis should shift to thinking creatively about how to best utilize social science expertise across an array of specific programmatic areas. Novel approaches within malaria control interventions are starting to emerge, such as integrating the distribution of insecticide-treated nets with measles immunizations. This same type of non-traditional thinking should be applied when developing career tracks for social scientists within Ministries of Health.

However, incentives for employment must rival or exceed those offered in other areas. Pay scales must be equitable and the value of multidisciplinary perspectives must be appreciated and desired by national malaria control programmes and Ministries of Health in order for career structures for long-term employment of social scientists to be established. For contractual employment, the same considerations should be shown. Short-term salaries for malaria-related projects should be competitive with salaries offered for similar work being conducted with other diseases, such as HIV/AIDS.

In the last five years, there has been a noticeable increase in the collaboration among social scientists and others engaged in malaria control. The interest in malaria by social scientists is clearly present. What are needed now are structures for training and employment that offer a professional career path over time.

## Authors' contributions

HAW and CJ obtained funding for the project. All authors contributed to the study conception and design. PN coordinated fieldwork, data collection and analysis with ongoing supervision from HAW and CJ. PN wrote the first draft of the article with critical revisions from HAW and CJ. PN, HAW, CJ, IN, SD and FG all read and approved the final manuscript.
